# Vitamins A, D, E, and K Intakes, Status, and Breastmilk Concentrations in Breastfeeding Women in Ireland

**DOI:** 10.1016/j.tjnut.2026.101611

**Published:** 2026-05-22

**Authors:** Malak Ali, Fiona O’Dwyer, Brona Murphy, Heleena Moni Bottu, Martina Rooney, Torres Sweeney, Sharleen L O’ Reilly, Aifric M O’Sullivan

**Affiliations:** 1 Institute of Food and Health, School of Agriculture and Food Science, University College Dublin, Dublin 4, Republic of Ireland; 2 Food for Health Ireland, University College Dublin, Dublin 4, Republic of Ireland; 3 School of Veterinary Medicine, University College Dublin, Republic of Ireland

**Keywords:** Breastmilk, lactation, vitamin A, vitamin D, vitamin E, vitamin K, metabolic health, maternal health, postpartum health

## Abstract

**Background:**

Fat-soluble vitamins A, D, E, and K are critical during the postpartum period for maternal recovery and infant development.

**Objectives:**

To determine the concentrations of vitamins A, D, E, and K in maternal serum and breastmilk during the early postpartum period in breastfeeding women.

**Methods:**

This was a secondary analysis of the WellFed 4-wk randomized controlled trial. Dietary recalls, serum, and breastmilk samples were collected from breastfeeding women at 4 and 8 wk postpartum (*n* = 48). Vitamin status and metabolic biomarkers were quantified using liquid chromatography–tandem mass spectrometry and a clinical analyzer, respectively. Associations were analyzed using correlation, regression, and mediation analysis (PROCESS macro for SPSS; IBM Corp).

**Results:**

Mean daily intakes for vitamins A, D, and E were 1230 ± 633 μg, 6.5 ± 4.6 μg and 13.3 ± 8.1 mg, respectively. All women were sufficient in serum retinol (2.1 ± 0.4, 2.1 ± 0.3 μmol/L), α-tocopherol (45.5 ± 9.5, 39.3 ± 8.7 μmol/L), and phylloquinone (1.0 ± 0.5, 1.1 ± 0.7 nmol/L) at weeks 4 and 8, respectively. Sixty-five percent of the cohort were vitamin D sufficient (61.7 ± 22.2 nmol/L, 56.7 ± 19.2 nmol/L). Vitamin A intake was positively associated with serum retinol concentrations (*r* = 0.386, *P* = 0.012, adjusted *P* = 0.048). Breastmilk retinol (3.7 ± 1.2, 3.2 ± 1.1 μmol/L) and α-tocopherol (15.3 ± 7.4, 12.4 ± 5.1 μmol/L) concentrations were adequate at both timepoints, 25(OH)D_2_ and 25(OH)D_3_ were below the analytical detection limit. Serum and breastmilk α-tocopherol were correlated (*r* = 0.363, *P* = 0.013, adjusted *P* = 0.026). Total cholesterol, triglycerides, and CRP predicted up to 56% of serum α-tocopherol variance.

**Conclusions:**

Maternal serum concentrations of vitamins A, E, and K are adequate, whereas approximately two-thirds of women are vitamin D sufficient. Breastmilk vitamins A and E are adequate and higher than previously reported, whereas vitamin D metabolites are undetectable. Dietary intake is positively associated with serum vitamins A and D, whereas serum vitamin E is associated with lipid-related biomarkers and correlated with breastmilk α-tocopherol, highlighting a potential link between maternal metabolic health and vitamin E in breastmilk.

This trial is registered at clinicaltrials.gov as NCT05924633 (https://clinicaltrials.gov/study/NCT05924633).

## Introduction

The postpartum period is a critical window for maternal recovery and infant development, as physiology and metabolism adjust after pregnancy and childbirth while providing essential nutrition through lactation [1]. The WHO recommend exclusive breastfeeding for the first 6 mo of life [2], highlighting the critical role of maternal nutrition in both short- and long-term health outcomes. Fat-soluble vitamins A, D, E, and K are particularly important during this period, as they support immune function, antioxidant defense, blood coagulation, and bone metabolism [[3], [4], [5], [6]]. Infants are born with low fat-soluble vitamin body stores due to limited placental transfer, which means they must depend on breastmilk to meet their early nutrient requirements [7]. Breastmilk fat-soluble vitamin concentrations vary widely, as they reflect not only dietary intake but also differences in maternal lipid metabolism, nutrient transport, and overall nutrition status [8,9]. Inter-individual variations in breastmilk composition, particularly during the early postpartum period, remain poorly understood. Women must replenish their own nutrient stores while producing milk for their infants, making it essential to understand the factors that influence vitamin concentrations in breastmilk to protect maternal and infant health.

Although vitamin D is tightly regulated metabolically, limited dietary intake and variable endogenous synthesis often result in insufficiency among lactating women and their infants [[10], [11], [12], [13]]. Postpartum physiological changes, inflammation, and altered lipid metabolism may further influence vitamin D transport and bioavailability, potentially affecting maternal status and transfer into breastmilk [14]. Vitamin D concentrations in breastmilk range from 0.25 to 2.0 μg/L, providing approximately 0.16 to 1.25 μg daily to exclusively breastfed infants—well below the recommended intake of 5 μg/d [[15], [16], [17]]. Although mean dietary vitamin D intake among pregnant women in Ireland is ∼10.8 μg/d [18], no data describing vitamin D intake among lactating women exist. Clinical trials show that intakes of 10 μg/d do not adequately increase breastmilk vitamin D concentrations, and maternal supplementation of ∼50 μg/d is required to improve infant vitamin D supply through lactation [17,19,20]. The limited dietary availability, coupled with the physiological demands of lactation, highlights the need to better understand maternal-infant vitamin D dynamics during the early postpartum period.

Vitamins A, E, and K share several physiological and nutrition features relevant to early lactation. Although maternal deficiencies are uncommon in well-nourished populations, concentrations of these vitamins in breastmilk vary widely among women, reflecting influences beyond dietary intake alone. Variations in lipid metabolism, inflammation, and maternal body composition can alter the mobilization and transport of retinol, α-tocopherol, and phylloquinone, thereby affecting their availability for secretion into milk [[21], [22], [23], [24], [25], [26], [27], [28]]. Vitamin A concentrations are generally positively associated with maternal serum status [29], whereas the relationships for vitamins E and K appear weaker or inconsistent, except when supplemented at high doses [30,31]. Notably, the Mothers, Infants and Lactation Quality Study (MILQ) Consortium does not provide reference values for vitamin K due to its consistently low concentrations in breastmilk [7]. The scarcity of data on vitamin K and the heterogeneity of findings for vitamins A and E highlight a shared gap in understanding how maternal nutrition and metabolic status impact the transfer of fat-soluble vitamins and therefore the concentrations in breastmilk during early lactation.

Despite the essential roles of fat-soluble vitamins in infant development, the determinants of their concentrations in breastmilk, particularly during the early postpartum period (2–8 wk) remain unclear. Given that maternal status, lipid metabolism, and physiological recovery may differentially influence vitamin transfer, further research is needed to clarify these relationships. The primary aim of this research is to quantify fat-soluble vitamin (A, D, E, and K) concentrations in maternal serum and breastmilk during the early postpartum period in breastfeeding women. An exploratory objective is to examine associations between fat-soluble vitamin concentrations, maternal dietary intake, and metabolic health markers.

## Methods

### Ethical approval

This study was carried out in accordance with the guidelines laid down in the Declaration of Helsinki. Ethical approval for the study was obtained through University College Dublin Human Research Ethics Committee (LS-23-07-OSullivan) and the National Maternity Hospital Research Ethics Committee (EC06.2023). All participants voluntarily gave both online and written informed consent before study participation. The study was registered at clinicaltrials.gov as NCT05924633.

### Study participant eligibility and recruitment

This study is a secondary analysis of data collected during the WellFed randomized, placebo-controlled trial, which recruited women between June 2023 and March 2024. Women were recruited from the community and National Maternity Hospital, Dublin, in 2 phases. In phase 1, women who were pregnant or up to 4 wk postpartum were recruited through online and poster advertisements and screened using an online questionnaire (SurveyMonkey Inc). Healthy, multiparous women aged 18 to 49 y with prior breastfeeding experience (>3 mo), pregnant with a healthy singleton pregnancy, met phase 1 inclusion criteria. Phase 2 recruitment and screening took place up to 4 wk postpartum, during which eligible women identified in phase 1 were contacted by phone and invited to participate. Women who were exclusively breastfeeding a healthy infant, free of breast or nipple infection, and not taking medications known to affect lactation met phase 2 criteria. Participants were excluded if they were aged <18 or >49 y at recruitment, first-time mothers, had <3 mo previous breastfeeding experience, smoked, were not independently living, unable to read, write, or understand English; followed a strict prescribed diet; had a milk protein allergy; or were in a COVID-19 very high-risk group.

### Study design

The WellFed randomized controlled trial (RCT) was a 4-wk double-blind randomized controlled trial (*n* = 58). The WellFed RCT sample size was determined using power calculations (α = 0.05, β = 0.20) informed by prior β-glucan studies, resulting in a target of 70 participants per group to allow for a 40% dropout rate. A researcher independent of the study randomized eligible participants to intervention (*n* = 30) and control (*n* = 28) groups using a computer-generated randomization schedule. The intervention group received a supplement containing a protein hydrolysate (LFC16) and yeast β-glucan (Wellmune). The control group received an identical maltodextrin capsule. Both groups were asked to take their capsule once daily for 4 wk. The study involved 2 home visits at week 4 and week 8 postpartum within the catchment counties of Dublin, Kildare, Meath, and Wicklow. The first visit took place within 4 wk and 6 d of the infant’s birth, the second visit occurred 4 wk later. Prior to both visits, participants were sent questionnaires including a dietary recall and a health and lifestyle questionnaire. Biological samples and anthropometric measurements were collected at week 4 and week 8 visits by trained researchers, according to standard operating procedures.

### Anthropometric measurements

Maternal anthropometric measurements were recorded at week 4 and week 8 visits. Height (Leicester Height Measure, Chasmors Ltd.), weight (Seca 803, Seca GmbH & Co.), and waist and hip circumference (Seca 201, Seca GmbH & Co.) were measured using standard operating procedures by trained researchers and with the participant wearing light clothing, no shoes, socks, belts or jewelry and with empty pockets.

### Dietary data collection and analysis

Dietary intake data were collected using Foodbook24, a validated self-administered online 24-h recall dietary assessment tool [32]. Food composition data on Foodbook24 are derived from the Food Composition Tables [33] and the Irish Food Composition Database [34]. The development and validation of Foodbook24 have been described in detail previously [32]. Participants were asked to complete three nonconsecutive 24-h recalls prior to each study visit. Women were excluded from the dietary analysis if they had only a single recall completed. Food and drink items were categorized into 59 different food groups in Foodbook24, which we subsequently collapsed into 24 groups based on compositional similarities. Mean intakes and percentage contributions of each food group to daily vitamins A, D, and E intakes were calculated. Vitamin K was not available from Foodbook24 due to limited vitamin K composition data currently available for foods in Ireland and Europe [35]. Vitamin D foods were classified as natural (e.g., fish, eggs) or fortified sources (e.g., fortified milk, cereal). Mean daily energy, nutrient (from foods and supplements), and food group intakes were calculated for the total population and consumers only. Under-reporting of energy intake was assessed by comparing reported energy intake to estimated basal metabolic rate using the Harris–Benedict equation [36]. A single participant was identified but because their inclusion did not alter results, they were retained in the final analyses. Nutrient adequacy was determined using the European Food Safety Authority dietary reference values, specifically the population reference intakes (PRIs) or adequate intakes for lactating women. Saturated fat intake was evaluated against WHO recommendations (<10% total energy) [37].

### Biological sample collection and processing

Participants collected a full breast expression using the Elvie electric breast pump (Elvie, Chiaro Technology Ltd) within 24-h prior to week 4 and week 8 morning home visits. They were instructed to gently swirl the breastmilk to ensure homogeneity before transferring 5 mL into a sterile plastic container; the remainder could be fed to the infant or stored for subsequent feeding. Samples were refrigerated (<4°C) at home prior to the visit. During home visits, maternal fasting blood samples (≥6 h fasted) were collected by a trained phlebotomist. Blood samples were left at room temperature for 30 min to coagulate, then placed on ice until processing in the laboratory. Breastmilk samples were kept on ice and immediately transported to the laboratory for processing. The total time between blood draw and laboratory processing was ∼1.5 h for all samples. In the laboratory, breastmilk samples were gently inverted, aliquoted into 1.5 mL samples and stored at −80°C until batch analysis at the trial completion. Blood samples were centrifuged for 15 mins at 1500 × *g* and 4°C and 500 μL of the serum layer was aliquoted and stored at −80°C until batch analysis.

### Biochemical analysis

Serum and breastmilk concentrations of vitamin A (all-trans retinol), D (25-hydroxyvitamin D_2_ and D_3_), and E (α- and γ*-*tocopherol) and K_1_ (phylloquinone) were analyzed at Bevital AS, Bergen, Norway (http://www.bevital.no) using authentic isotope-labeled internal standards and liquid chromatography–tandem mass spectrometry (LC-MS/MS) as previously described [38]. Breastmilk sample preparation followed an established protocol [38], with the exception of vitamin A, which included an additional saponification step prior to the standard sample preparation. Briefly, this included mixing 50-μL breastmilk with 50 μL of ethanol, and 50 μL of 17.82 mol/L potassium hydroxide, and the internal standard (^2^H_6_-all-trans retinol), before incubating at 55°C for 11 h. Quality and accuracy were monitored using internal quality controls and through participation in the Vitamin D and K External Quality Assessment Schemes. Limits of detection were: all-trans retinol, 0.1 μmol/L; 25-hydroxyvitamin D_2_, 5nmol/L and D_3_, 3.3 nmol/L; *α*-, 2 μmol/L and *γ-*tocopherol, 0.1 μmol/L; and phylloquinone, 0.5 nmol/L. Within- and between-day coefficient of variation ranged from 2.3% to 6.6% and 3.6% to 8.1%, respectively. Vitamin A status was classed as deficient (<0.7 μmol/L), insufficient (0.7–1.05 μmol/L), or sufficient (≥1.05 μmol/L) based on serum retinol concentrations [39]. Vitamin D status was defined following Institute of Medicine guidelines as deficient (<30 nmol/L), insufficient (30–50 nmol/L), or sufficient (≥50 nmol/L) based on serum 25(OH)D [40]. Vitamin E status was considered low if serum α-tocopherol concentrations were <12 μmol/L [41]. There are no universally agreed cut-offs for vitamin K1 status; however, concentrations between 0.22 and 3.95 nmol/L are generally considered within the normal range for healthy adults [42].

Serum concentrations of blood total cholesterol, HDL cholesterol, triglycerides, C-reactive protein (CRP), and glucose were assessed using a HORIBA Pentra C400 (Randox) with the appropriate reagent kits and following the manufacturer’s instructions. Quality and accuracy were monitored using internal quality controls, in accordance with the manufacturer’s guidelines, with inter- and intra-assay coefficients of variation maintained <4.5%.

### Statistical analysis

Statistical analysis was performed in SPSS for Macintosh (version 29; IBM Corp). Missing data were minimal, therefore complete-case analysis was performed. Continuous data are presented as mean ± SD and categorical data as count and percentage. Normality was assessed using histograms, Q-Q plots, and the Shapiro–Wilk test, with *P* ≤ 0.05 suggesting non-normality. Non-normal variables were transformed using logarithmic or square-root functions. Two-way mixed analysis of variance compared changes in breastmilk and serum nutrient concentrations and serum health biomarkers from week 4 to week 8. Pearson’s correlation coefficients assessed associations between breastmilk and serum nutrient concentrations, dietary intakes (nutrient and food groups, g/day), and health biomarkers. Energy intake and BMI (kg/m^2^) were identified as covariates using correlation analysis. For dietary associations, each nutrient and its top 3 contributing food groups (based on percentage contribution to total intake) were selected as candidate predictors. Significant correlations (*P* < 0.05) were visually inspected using scatter plots and fitted regression lines were inspected for linearity. To test the robustness of associations, correlations were repeated excluding nonconsumers of food groups; only associations that remained significant were retained for further analysis. Variables showing evidence of association after adjustment for multiple comparisons were entered into stepwise linear regression models to explore potential predictors of serum and breastmilk nutrient concentrations. Where significant serum-breastmilk nutrient associations persisted, mediation analyses were performed using the PROCESS macro (version 5.0) for SPSS (IBM Corp) to examine whether dietary intake mediated these relationships [43]. Associations between serum nutrient concentrations and health biomarkers were explored using the same approach. Post hoc adjustment for multiple comparisons was performed using the Benjamini–Hochberg procedure to control the false discovery rate at 0.05; only associations that remained significant after this correction were interpreted as statistically significant. Statistical significance was set at *P* < 0.05.

Recognizing the original RCT study design, we did some preliminary data analysis to justify the approach for this article. There was no group∗time interaction or main effect of intervention treatment group or time for dietary intake data. Mean daily nutrient and food group intakes were calculated using all 24-h recalls collected at week 4 and week 8 to provide a more accurate estimate of habitual intakes. There was no group∗time interaction or main effect of intervention treatment group on serum or breastmilk vitamin A, D, E, or K concentrations or serum health biomarkers and so intervention treatment group was not included in this analysis. There was a significant effect of time on serum and breastmilk vitamin A, D, E, and K concentrations and serum health biomarkers, therefore, serum and breastmilk results are presented for week 4 and week 8 separately.

## Results

### Demographics

**FIGURE 1:**
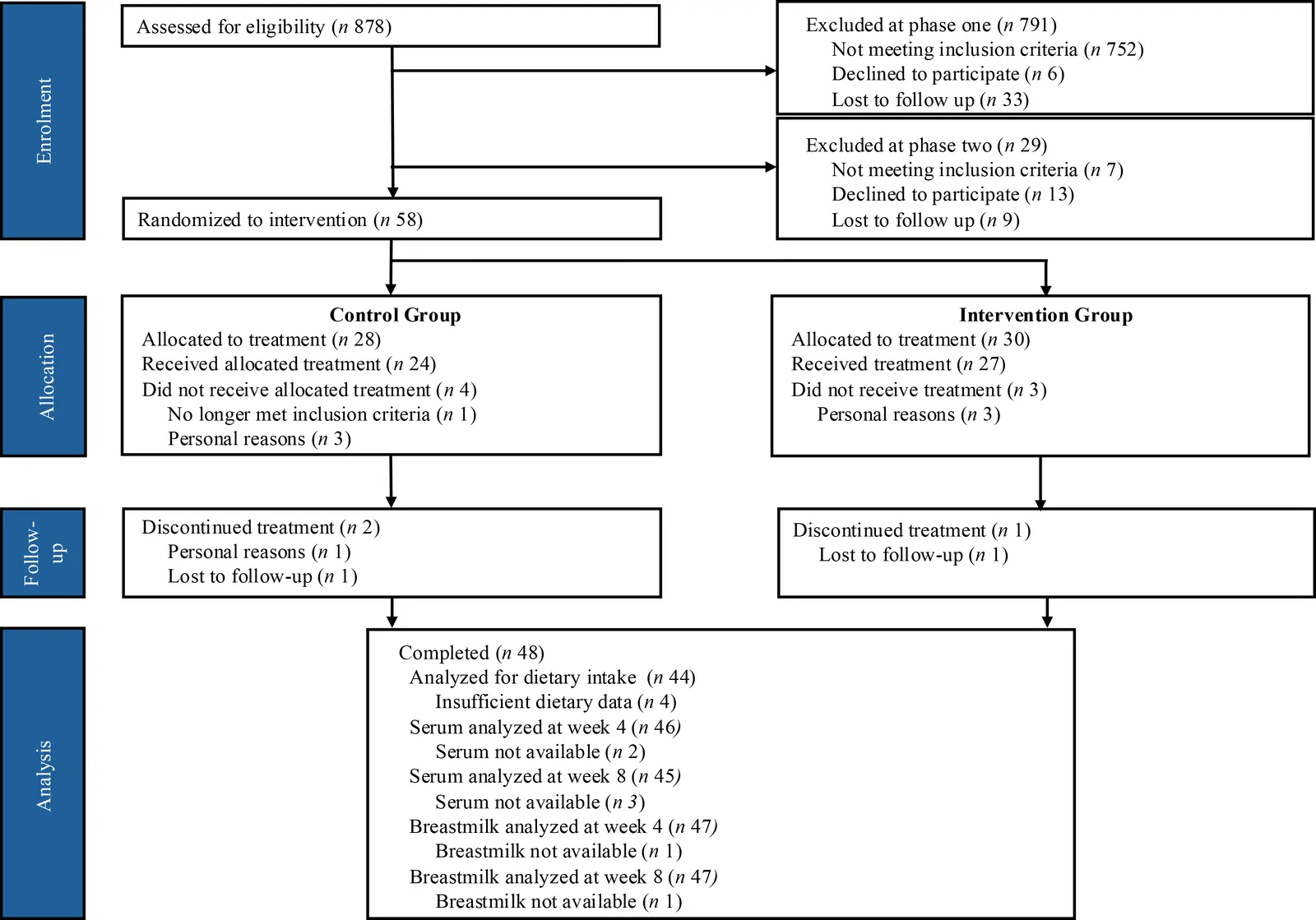
CONSORT flow diagram of study participants [44]. Intervention and control group data were merged for the purpose of this secondary analysis.

Fifty-eight participants were randomized, and 51 started the study and received the allocated treatment. Three participants were lost to follow-up (illness, family reasons, loss of interest) and were not included in the analysis, and 48 completed the study (Figure 1) [44]. Data collected from 48 women aged 37.5 ± 3.6 y were available for this secondary analysis (Table 1). Ninety-eight percent of participants self-reported as White, all had completed a third-level (university/college) degree, and 64% reported they consumed dietary supplements, including multivitamins, vitamin D, omega-3, iron, and protein supplements. The most consumed supplements were Vitabiotics Pregnacare and Pregnacare Breastfeeding multivitamins (Vitabiotics Ltd.). These supplements provide 10-μg vitamin D, 4–20 mg α-tocopherol equivalents, and 70-μg vitamin K per daily dose.

**TABLE 1:** Maternal characteristics (*n =* 48).

Characteristic	Mean	SD
Maternal age (y)	37.5	3.6
Maternal weight (kg)	74.0	12.9
Maternal height (cm)	166.2	6.3
Maternal BMI (kg/m^2^)	27.0	4.4
Maternal race (% White)	98.0	
Education level (% 3^rd^ level)^1^	100.0	
Dietary supplement user (% yes)	64.0	

All values are presented as mean and SD unless indicated otherwise stated.

1 Third level education refers to university/college level education.

### Nutrient status and breastmilk nutrient concentrations

Serum samples were available for 46 and 45 women at week 4 and 8, respectively; 43 serum samples were available for paired analysis. Paired breastmilk samples were available for 47 women. Maternal serum and breastmilk retinol, 25(OH)D_2_, 25(OH)D_3_, α-tocopherol, γ-tocopherol, and phylloquinone concentrations at week 4 and week 8 are presented in Table 2 [[39], [40], [41]]. All participants were classified as sufficient for serum retinol (2.1 ± 0.4, 2.1 ± 0.3 μmol/L), α-tocopherol (45.5 ± 9.5, 39.3 ± 8.7 μmol/L), and phylloquinone (1.0 ± 0.5, 1.1 ± 0.7 nmol/L) concentrations at 4 weeks and 8 weeks, respectively, despite a significant decrease in α-tocopherol (*P* < 0.001). Sixty-five percent of women were classified as vitamin D sufficient based on serum 25(OH)D_3_ concentrations >50 nmol/L. Breastmilk retinol and α-tocopherol concentrations declined significantly over time (retinol: 3.7 ± 1.2 μmol/L, 3.2 ± 1.1 μmol/L, *P* = 0.017; α-tocopherol: 15.3 ± 7.4 μmol/L, 12.4 ± 5.1 μmol/L, *P* = 0.018). Breastmilk 25(OH)D_2_ and 25(OH)D_3_ concentrations were below the limit of detection throughout (<5 and 3.3nmol/L, respectively). The increase in serum and breastmilk α-tocopherol remained significant after controlling for multiple comparisons using the Benjamini–Hochberg procedure (adjusted *P* < 0.001 and adjusted *P* = 0.036).

**TABLE 2:** Serum and breastmilk nutrient concentrations at 4 and 8 wk postpartum.

Biomarker	Week 4		Week 8		*P* value
	Mean	SD	Mean	SD	
Serum (*n* = 43)					
Retinol (μmol/L)	2.1	0.4	2.1	0.3	0.328
25(OH)D_3_ (nmol/L)	61.7	22.2	56.7	19.2	0.148
25(OH)D_2_ (nmol/L)^1^	6.2	1.2	6.2	1.4	0.595
α-tocopherol (μmol/L)	45.5	9.5	39.3	8.7	<0.001
γ-tocopherol (μmol/L)	2.6	0.8	2.6	0.7	0.905
Phylloquinone (nmol/L)^2^	1.0	0.5	1.1	0.7	0.521
Breastmilk (*n* = 47)					
Retinol (μmol/L)	3.7	1.2	3.2	1.1	0.017
25(OH)D_3_ (nmol/L)	<LOD	—	<LOD	—	—
25(OH)D_2_ (nmol/L)	<LOD	—	<LOD	—	—
α-tocopherol (μmol/L)	15.3	7.4	12.4	5.1	0.018
γ-tocopherol (μmol/L)	1.8	0.9	1.7	0.8	0.454
Phylloquinone (nmol/L)	4.6	2.8	4.0	2.7	0.150

All values are presented as mean and SD.

Repeated measures general linear model, was used to determine changes in biomarker concentrations. *P* < 0.05 considered statistically significant. The decrease in serum and breastmilk α-tocopherol remained significant after controlling for multiple comparisons using the Benjamini–Hochberg procedure (adjusted *P* < 0.001 and adjusted *P* = 0.036).

In breastmilk, 25(OH)D_2_ and 25(OH)D_3_ were below the limit of detection of 5.0 nmol/L and 3.3 nmol/L, respectively and are presented as (<LOD). Reference threshold for adequacy in serum samples were defined as follows: 25(OH)D ≥50 nmol/L; retinol >1.05 μmol/L; α-tocopherol ≥12μmol/L [[39], [40], [41]].

25(OH)D, 25-hydroxyvitamin D; LOD, limit of detection.

1 25(OH)D_2_ detectable in week 4 (*n* = 10) and week 8 (*n* = 13).

2 Phylloquinone detectable in week 4 (*n* = 41) and week 8 (*n* = 37).

### Macro and micronutrient intakes

Forty-four women completed at least 2 dietary recalls. Mean daily energy intake was 2228 ± 477kcal (Table 3). The proportion of energy from protein, fat and carbohydrates were 16.2% ± 3.1%, 38.8% ± 4.8%, and 46.5% ± 5.8% respectively. Eighty percent of participants exceeded fat intake recommendations, and 95% exceeded WHO saturated fat recommendations. Mean dietary fiber intake was 19.8 ± 5.1 g/d, with 14% of participants reaching the EFSA recommendation of 25 g/d. Mean daily intakes of vitamin B_12_, calcium and iron were above European Food Safety Authority adequate intakes and PRIs with 50%, 61%, and 39% of participants meeting requirements, respectively. Mean daily vitamin C intake was below EFSA PRIs at 105.4 ± 67.1 mg with only 21% of participants meeting requirements (Table 3).

**TABLE 3:** Mean daily intake of macro and micronutrients and percentage of participants meeting EFSA DRVs^1^ (*n* = 44).

Nutrient	Mean	SD	Meeting ESFA/WHO DRVs % (*n*)	EFSA/WHO DRV
Energy (kcal)	2228.0	477.0		
Protein (g)	89.9	24.8	66 (29)	0.83/kg+19g
Protein (%TE)	16.2	3.1		
Carbohydrates (g)	258.0	58.4		
Carbohydrates (%TE)	46.5	5.8	64 (28)	45%–60% TE
Fat (g)	97.1	27.4		
Fat (%TE)	38.8	4.8	20 (9)	20%–35% TE
Dietary fiber (g)	19.8	5.1	14 (6)	25.0
Saturated fat (g)	38.9	12.8		
Saturated fat (%TE)	15.5	3.1	5 (2)	<10% TE
Vitamin A (μg RE)	1230.0	633.0	39 (17)	1300.0
Vitamin D (μg)	6.5	4.6	4 (2)	15.0
Vitamin E (mg)	13.3	8.1	48 (21)	11.0
Vitamin B_12_ (μg)	6.0	4.6	50 (22)	5.0
Vitamin C (mg)	105.0	67.1	21 (10)	155.0
Calcium (mg)	1012.0	287.0	61 (27)	950.0
Iron (mg)	16.3	9.3	39 (17)	16.0

Abbreviations: DRV, dietary reference value; EFSA, European Food Safety Authority; RE, retinol equivalents; TE, percent of total energy.

All values are presented as mean and SD.

Vitamin K intake data were unavailable from Foodbook24 due to limited composition data in Irish and European databases [35]. Mean daily nutrient intakes from participants who completed ≥2 recalls (*n* = 44).

1 Intakes include contributions from both food and dietary supplements.

### Dietary intake data for vitamin A, D and E

Vitamin A intake from food and supplements was 1230 ± 633 μg/d, expressed as retinol equivalents, with 39% of participants meeting EFSA intake recommendations (Table 3). Supplements contributed 126 ± 174 μg/d to total vitamin A intake. Among the main dietary contributors to vitamin A intake were vegetables and salads (391 ± 351 μg/d; 29% ± 22%), soups and sauces (163 ± 256 μg/d; 12% ± 15%), and butter, spreads and oils (130 ± 110 μg/d; 11% ± 9%) (Table 4). Vitamin D intake from food and supplements was 6.5 ± 4.6 μg/d, with 96% of all participants consuming less than the current EFSA recommendation of 15μg/d (Table 3). Supplements contributed 3.0 ± 3.6 μg/d to total vitamin D intake. The top 4 food groups contributing to vitamin D intake were eggs and egg dishes (0.8 ± 1.0 μg/d; 13% ± 6%), fish and fish products (0.7 ± 1.2 μg/d; 11% ± 18%), fortified breakfast cereals (0.5 ± 1.0 μg/d; 8% ± 10%), and milk and yogurt (0.3 ± 0.7 μg/d; 6% ± 12%) (Table 4). Overall, natural sources of vitamin D accounted for 63% of total intake, whereas fortified foods contributed the remaining 37%. The mean daily intake of vitamin E from food and supplements was 13.3 ± 8.1 mg/d with 48% of participants meeting the dietary intake recommendations (Table 3). Supplements contributed 6.3 ± 8.6 mg/d to total vitamin E intake. Among the top dietary contributors to vitamin E intake were grains, rice, pasta, and savories (1.3 ± 1.0 mg/d; 12% ± 10%); meat dishes (1.0 ± 0.9 mg/d; 7% ± 6%); and sugars, confectionery and preserves (1.2 ± 1.0 mg/d; 9% ± 8%) (Table 4).

**TABLE 4:** Mean daily intake and percentage contribution of food groups to total vitamin A, D, and E intakes (*n* = 44).

Food Group	Weight (g)		Vitamin A (μg/d)		Vitamin A %		Vitamin D (μg/d)		Vitamin D %		Vitamin E (mg/d)		Vitamin E %	
	Mean	SD	Mean	SD	Mean	SD	Mean	SD	Mean	SD	Mean	SD	Mean	SD
Milk and yogurt	114.0	94.6	56.7	67.8	5.1	6.4	0.3	0.7	6.2	12.4	0.4	0.7	2.6	3.8
Breakfast cereals	95.8	91.1	21.8	33.5	1.7	2.4	0.5	1.0	7.0	10.4	0.5	0.5	4.5	3.8
Fish and fish products	24.2	28.0	9.4	15.7	0.8	1.5	0.7	1.2	10.5	17.6	0.6	0.5	5.1	4.2
Eggs and egg dishes	24.9	31.3	54.3	39.8	5.4	4.6	0.8	1.0	13.3	16.2	0.6	0.5	5.9	5.5
Meat dishes	141.0	97.8	72.8	86.3	6.4	5.6	0.2	0.2	3.5	5.1	1.0	0.9	7.6	6.3
Grains, rice, pasta and savories	135.0	78.9	62.8	97.5	5.0	4.9	0.2	0.3	7.2	9.9	1.3	1.0	11.6	10.3
Sugars, confectionery and preserves	42.9	29.2	17.2	15.8	1.7	1.9	0.01	0.01	0.7	4.0	1.2	1.0	9.3	8.1
Vegetables and salads	65.5	51.1	391.0	351.0	29.1	22.0	0.0	0.01	0.3	1.6	0.6	0.6	5.6	5.0
Soups and sauces	59.2	56.7	163.0	256.0	11.5	15.2	0.01	0.05	0.2	1.0	1.0	1.1	6.3	6.9
Butter, spreads and oils	12.4	9.3	130.0	110.0	11.4	9.1	0.1	0.1	3.8	7.6	0.3	0.3	2.7	3.1

All values are presented as mean and SD.

Vitamin K intake data were unavailable from Foodbook24 due to limited composition data in Irish and European databases [35].

### Health biomarkers

Concentrations of total cholesterol, triglycerides, HDL cholesterol, CRP, and glucose at week 4 and week 8 are presented in Table 5. There was a decrease in total cholesterol (6.5 ± 1.3, 5.9 ± 1.1 mmol/L, *P* < 0.001), triglycerides (0.6 ± 0.2, 0.5 ± 0.2 mmol/L, *P* = 0.035), and CRP (5.8 ± 12.5, 1.2 ± 2.3 mmol/L, *P* = 0.023) from week 4 to week 8. The decrease in total cholesterol remained significant after controlling for multiple comparisons using the Benjamini–Hochberg procedure (adjusted *P* < 0.001). HDL cholesterol and glucose concentrations did not change over time.

**TABLE 5:** Biomarkers of metabolic health at weeks 4 and 8 postpartum (*n* = 43).

Biomarker	Week 4		Week 8		*P* value
	Mean	SD	Mean	SD	
Total cholesterol (mmol/L)	6.5	1.3	5.9	1.1	<0.001
Triglycerides (mmol/L)	0.6	0.2	0.5	0.2	0.035
HDL (mmol/L)	2.1	0.5	2.1	0.4	0.881
C-reactive protein (mg/L)	5.8	12.5	1.2	2.3	0.023
Glucose (mmol/L)	5.8	0.5	5.7	0.5	0.199

All values are presented as mean and SD.

Repeated measures general linear model was used to determine changes in biomarker concentrations. *P* < 0.05 considered statistically significant. The decrease in total cholesterol remained significant after controlling for multiple comparisons using the Benjamini–Hochberg procedure (adjusted *P* < 0.001).

### Serum retinol, 25(OH)D_3_ and α-tocopherol concentrations and dietary intake

At week 4, serum retinol concentrations were positively correlated with vitamin A intake (*r* = 0.386, *P* = 0.012, adjusted *P* = 0.048) and butter, spread, and oil intakes (*r* = 0.373, *P* = 0.015, adjusted *P* = 0.030). Vitamin A intake was retained in the final stepwise linear regression model (β = 0.386, *P* = 0.012), explaining 12.8% of the variance in serum retinol concentrations [F(1,40)=7.01, *P* = 0.012, adjusted R^2^ = 0.128]. At week 8, serum 25(OH)D concentrations were positively associated with vitamin D intake (*r* = 0.345, *P* = 0.027, adjusted *P* = 0.108). Serum α-tocopherol and vitamin E intake or any food group were not correlated at any time point.

### Serum and breastmilk retinol, 25(OH)D_3_, α- and γ-tocopherol and phylloquinone concentrations

At week 4, serum α-tocopherol was correlated with breastmilk α-tocopherol (*r* = 0.363, *P* = 0.013, adjusted *P* = 0.026). Mediation analyses indicated a significant direct relationship between serum and breastmilk α-tocopherol (B = 0.27, 95% CI: 0.05, 0.50, *P* = 0.018) in a model that included vitamin E intake. The indirect effects through vitamin E intake were not significant, as the confidence intervals included zero (B = −0.001, 95% CI: −0.055, 0.018). Serum and breastmilk retinol or phylloquinone were not correlated.

### Health biomarkers and serum retinol, 25(OH)D_3_, α- and γ-tocopherol and phylloquinone concentrations

At week 4, serum retinol was positively correlated with triglycerides (*r* = 0.386, *P* = 0.010, adjusted *P* = 0.050) and total cholesterol (*r* = 0.378, *P* = 0.012, adjusted *P* = 0.030). Triglycerides were retained in the final stepwise linear regression model (β = 0.386, *P* = 0.015), explaining 12.8% of the variance in serum retinol concentrations [F(1,40)=7.02, *P* = 0.011, adjusted R^2^ = 0.128] (Table 6). At week 8, serum retinol was positively correlated with triglycerides (*r* = 0.342, *P* = 0.023, adjusted *P* = 0.105). At week 4, serum α-tocopherol was positively correlated with triglycerides (*r* = 0.550, *P* = <0.001, adjusted *P* < 0.001) and total cholesterol (*r* = 0.718, *P* < 0.001, adjusted *P* < 0.001). Triglycerides and total cholesterol were retained in the final stepwise linear regression model (β = 0.293 and β = 0.591, respectively), explaining 56% of the variance in serum α-tocopherol concentrations [F(1,40)=42.55, *P* < 0.001, adjusted R^2^ = 0.564] (Table 6). At week 8, serum α-tocopherol was positively correlated with triglycerides (*r* = 0.351, *P* = 0.020, adjusted *P* < 0.001), CRP (*r* = 0.396, *P* = 0.008, adjusted *P* = 0.020), total cholesterol (*r* = 0.631, *P* < 0.001, adjusted *P* = 0.033), and glucose (*r* = 0.325, *P* = 0.031, adjusted *P* = 0.039). CRP and total cholesterol were retained in the final model of a stepwise linear regression (β = 0.355 and β = 0.607, respectively), explaining 50% of the variance in serum α-tocopherol concentrations (F(2,41)=22.56, *P* < 0.001, adjusted R^2^ = 0.501). At week 8, serum γ-tocopherol was positively correlated with triglycerides (*r* = 0.517, *P* = 0.002, adjusted *P* = 0.002). Triglycerides were retained in the stepwise linear regression model (β = 0.517, *P* < 0.001), explaining 25% of the variance in serum γ-tocopherol concentrations (F(1,42)=15.3, *P* < 0.001, adjusted R^2^ = 0.250) (Table 6). At week 8, serum phylloquinone was positively correlated with HDL cholesterol (*r* = 0.440, *P* = 0.030, adjusted *P* = 0.150). Serum 25(OH)D was not correlated with any health biomarkers at any time point.

**TABLE 6:** Stepwise linear regression models predicting week 4 and week 8 serum retinol, α-tocopherol, and γ-tocopherol concentrations from health biomarkers (*n =* 43)^1^.

Biomarker	Week 4				Week 8			
	B	β	SE	*P* value	B	β	SE	*P* value
Retinol								
Triglycerides (mmol/L)	0.399	0.386	0.150	0.011	—	—	—	—
α-tocopherol								
Total cholesterol (mmol/L)	4.378	0.591	0.849	<0.001	4.720	0.607	0.840	<0.001
Triglycerides (mmol/L)	8.241	0.293	3.223	0.015	—	—	—	—
C-reactive protein (mg/L)	—	—	—	—	2.152	0.355	0.655	0.002
γ-tocopherol								
Triglycerides (mmol/L)	—	—	—	—	1.082	0.517	0.277	<0.001

β, standardized regression coefficient; B, unstandardized regression coefficient; SE.

Multiple linear regression using the “stepwise” method in SPSS was used to determine predictors of serum nutrient concentrations. *P* < 0.05 considered statistically significant.

1 Predictors correspond to the timepoint of the outcome variable.

## Discussion

This study reports adequate serum and breastmilk retinol and α-tocopherol concentrations despite less than half of women meeting EFSA recommendations for dietary intake. In contrast, 2 in 3 women had sufficient vitamin D status; only 4% met dietary requirements, and breastmilk concentrations were undetectable, raising concerns for women and infants. The clinical biochemistry at 4 wk postpartum highlights the metabolic challenge that pregnancy brings, although elevated biomarkers were lower at week 8. Although associations between serum α-tocopherol, total cholesterol, and triglycerides are as expected, it is unclear why we see a positive relationship with inflammatory marker CRP in this cohort. These findings highlight a disconnect between fat-soluble vitamin intakes, status, and transfer to breastmilk, as well as highlight a major gap in our understanding of fat-soluble vitamin metabolism during lactation as well as its association with metabolic health.

Vitamin D deficiency remains a widespread global health concern and even more so for lactating women given increased physiological demands in the postpartum period as well as the requirement for breastmilk synthesis. Although two-thirds of this cohort were classified as vitamin D sufficient, which is higher than what others report during pregnancy [45] and postpartum [46], the major concern in this cohort is the fact that breastmilk concentrations were undetectable. The breastmilk matrix presents analytical challenges even with advanced technologies like LC-MS/MS; however, low breastmilk vitamin D concentrations are known [[47], [48], [49]]. As a result, some countries, including Ireland, mandate all breastfed infants to receive a daily supplement of 5-μg vitamin D up to 12 mo [50]. Like the disconnect between vitamin D serum status and breastmilk concentrations, dietary intake was also low and explained <10% of the serum 25OHD concentration variation at week 8. Almost all women did not meet recommendations (96%), and the majority of vitamin D (67%) came from natural foods such as fish, eggs, and dairy, which presents another concern for this cohort. Although dietetic management will take a “food first” approach to meet nutrient requirements, RCT evidence shows that supplementation as high as 50 μg/day is required to ensure adequate infant vitamin D supply through breastmilk [19,20]. Furthermore, the current environmental context is forcing a reduction in animal-source foods to meet sustainability targets that will increase vitamin D intake shortfalls if not compensated through supplementation or fortified foods. Indeed, vegetarian and vegan lactating women have lower vitamin D intakes and breastmilk concentrations (1.10 μg/L) compared with omnivorous women (3.43 μg/L) [51]. In summary, these findings reemphasize some knowns with respect to low maternal vitamin D intakes, status and breastmilk concentrations but also highlight some critical unknowns with respect to the disconnect between maternal vitamin D status and breastmilk concentrations.

All women were sufficient for serum retinol, α-tocopherol, and phylloquinone, as well as breastmilk concentrations of retinol, α-tocopherol, and phylloquinone were higher than those described in earlier studies [27,52,53]. A multinational cross-sectional study of lactating women in Australia, Canada, Chile, China, Japan, Mexico, the Philippines, the United Kingdom, and the United States reported retinol concentrations between 1.0 and 1.6 μmol/L [52] relative to approximately 3.5 μmol/L in this study. Similarly, a recent meta-analysis reported breastmilk α-tocopherol concentrations between 9.4 and 7.6 μmol/L for transitional and mature milk, respectively [27], relative to a range of 15.3 to 12.4 μmol/L between week 4 and week 8 in this study. The disconnect with dietary intake remains; despite sufficient status, less than half of women were meeting dietary requirements for vitamin A and E (vitamin K data not available), and associations with nutrient intakes were weak (vitamin A) or nonexistent (vitamin E). Furthermore, mediation analysis showed that the association between serum and breastmilk α-tocopherol was independent of vitamin E intake. Although there is less published data with respect to the serum-breastmilk link, there is some evidence in the literature to support this observation [[54], [55], [56]]. The positive take-home from this analysis is that breastmilk concentrations of vitamins A and E in well-nourished women are well above infant requirement thresholds, reinforcing the critical message that breastmilk is the reference standard for infant feeding; however, the significant gaps remain in our understanding of the relationship between dietary intakes, circulating status, and breastmilk concentrations.

Metabolic health in the postpartum period is an aspect of maternal health that is often overlooked. Pregnancy and childbirth present significant metabolic challenges, placing women at risk for negative pregnancy outcomes and potential long-term impacts on health [57]. Total cholesterol, triglycerides, and CRP were all high at week 4, and decreased by week 8 postpartum, although they did not reach healthy ranges within that timeframe. As expected, serum α-tocopherol was positively associated with total cholesterol and triglycerides, which together explained 56% of variance in week 4 α-tocopherol. However, because vitamin E is transported in circulating lipoproteins, the associations observed between α-tocopherol and lipid markers likely reflect lipoprotein transport rather than metabolic regulation. Although these associations are well established among nonpregnant adults, with numerous studies demonstrating that α-tocopherol concentrations are associated with circulating lipids [58,59], there is a notable gap in the literature regarding whether these relationships persist during the postpartum period. To our knowledge, no study has previously examined these associations in early postpartum lactating women, a population in which lipid metabolism undergoes substantial changes. Conversely, the positive association between serum α-tocopherol and CRP concentrations was not expected. Research suggests an inverse relationship exists between serum α-tocopherol and CRP and other markers of inflammation [59,60]; however, to our knowledge, no study has reported this relationship in the early postpartum period. Given that α-tocopherol is transported via lipoproteins, serum concentrations may not only reflect dietary intake but also the broader physiological changes that occur in the postpartum period. These complex interrelationships are important to consider when interpreting vitamin E status, as elevated concentrations may reflect postpartum changes in lipid metabolism and body composition adaptations rather than actual nutritional sufficiency. These findings highlight the unique metabolic profile of the early postpartum period and underscore the need for further research to clarify the mechanisms underlying the observed associations between serum α-tocopherol and metabolic and inflammatory markers.

### Strengths and limitations

To our knowledge, this is the first study in Ireland to simultaneously assess maternal serum and breastmilk concentrations of retinol, 25(OH)D, tocopherol, and phylloquinone postpartum. Maternal dietary intake was assessed using a validated 24-h recall, with procedures to minimize under-reporting. However, vitamin K intake data were unavailable due to limited compositional information, preventing analysis of relationships with serum and breastmilk phylloquinone. A key strength of this research is the use of isotope-labeled internal standards with LC-MS/MS, the reference standard for micronutrient analysis, enabling comparability with other studies. However, the multiplex assay may lack sensitivity to detect the very low concentrations of vitamin D in breast milk; levels were below the limit of detection, likely reflecting assay limitations rather than true absence. Singleplex assays with lower detection limits may improve quantification in future studies. As a secondary analysis of a trial not originally designed to assess fat-soluble vitamins, findings should be interpreted as associative rather than causal or mechanistic. Analyses were conducted on complete cases, and bias due to nonrandom missing data cannot be excluded. Additionally, detailed information such as supplement use, supplement composition, and maternal diet during pregnancy, and sunlight exposure was not collected, which may limit the interpretation of intake-status relationships. The relatively homogeneous cohort (White, highly educated, older maternal age) may restrict generalizability, particularly for vitamin D, given the influence of skin pigmentation and genetics on its metabolism [61,62]. The small sample size may have limited statistical power and increased the risk of residual confounding; therefore, these findings should be interpreted with caution and confirmed in larger studies. Regression analyses should be considered exploratory and hypothesis-generating. Despite the small sample size, this study provides valuable data on maternal and breastmilk concentrations of retinol, 25(OH)D, tocopherols, and phylloquinone during lactation.

This study provides novel insights into postpartum fat-soluble vitamin status and metabolism, representing the first study in Ireland and one of few in Europe to concurrently assess maternal serum and breastmilk concentrations of retinol, 25(OH)D, α-tocopherol, and phylloquinone using LC-MS/MS. Overall, maternal status for vitamins A, E, and K is adequate, and breastmilk concentrations are higher than previously reported, reflecting the nutrient quality of breastmilk in this cohort. In contrast, persistently low vitamin D intakes and undetectable breastmilk concentrations highlight an urgent need for improved strategies to support maternal and infant vitamin D sufficiency, particularly as shifts toward more plant-based diets may further reduce intake from animal-source foods. The observed relationships between α-tocopherol and metabolic markers point to complex physiological adaptations in the early postpartum period that warrant further study. Collectively, these findings advance understanding of how maternal nutrition and metabolism influence nutrient transfer to breastmilk and emphasize the need for targeted nutrition guidance and policy actions to optimize maternal recovery and infant health.

## Author contributions

The authors’ contributions were as follows: AOS, SOR, and TS designed the research. MA, BM, FOD, HMB, and MR conducted the research. MA and AOS analyzed the data and wrote the article. AOS has primary responsibility for the final content. All authors reviewed, edited and approved the final paper.

## Data availability

Data described in the paper, code book, and analytic code will not be made available due to ethical restrictions and the terms of consent.

## Funding

This study was supported by Food for Health Ireland, from Enterprise Ireland (TC-2018-0025 to AOS, SOR and TS). MAs PhD was supported by Department of Agriculture, Food and the Marine, Ireland (2021R610). The funders had no role in the analyses or interpretation of data, in the writing of the paper or in the decision to publish the findings.
